# Case report: Early-onset parkinsonism among the neurological features in children with *PHACTR1* variants

**DOI:** 10.3389/fneur.2023.1181015

**Published:** 2023-07-06

**Authors:** Roberto Previtali, Alessia Leidi, Martina Basso, Giana Izzo, Cecilia Stignani, Luigina Spaccini, Maria Iascone, Pierangelo Veggiotti, Stefania Maria Bova

**Affiliations:** ^1^University of Milan, Milan, Italy; ^2^Department of Pediatric Radiology and Neuroradiology, V. Buzzi Children's Hospital, Milan, Italy; ^3^Department of Pediatric Orthopedics, V. Buzzi Children's Hospital, Milan, Italy; ^4^Clinical Genetics Unit, Department of Obstetrics and Gynecology, V. Buzzi Children's Hospital, University of Milan, Milan, Italy; ^5^Molecular Genetics Section, Medical Genetics Laboratory, Papa Giovanni XXIII Hospital, Bergamo, Italy; ^6^Department of Biomedical and Clinical Sciences, L. Sacco, University of Milan, Milan, Italy; ^7^Pediatric Neurology Unit, Vittore Buzzi Children's Hospital, Milan, Italy

**Keywords:** *PHACTR1*, parkinsonism, movement disorder, epilepsy, developmental delay

## Abstract

*PACHTR1* is expressed in cardiovascular and neurological tissues. In the brain, it has a role in pre- and post-natal maturation. Previously reported *PHACTR1*-mutated patients showed early-onset epilepsy and intellectual disability. We describe two unreported cases with *de novo* pathogenic variants in *PHACTR1* and their clinical pictures, compared with those of cases already reported in the literature. In line with previous reports, the two patients presented early-onset developmental and epileptic encephalopathy. In addition, one patient developed a speech disorder and a progressive movement disorder characterized by hypertonus, hypo-bradykinesia, hypomimia, ataxic gait, and retropulsion. She was treated with levodopa without any clinical improvement. Pathogenic variants in *PHACTR1* may result in a cardiological or neurological phenotype. Severe developmental delay, intellectual disability, and early-onset developmental and epileptic encephalopathy are the main features of *PHACTR1*-mutated patients with neurological involvement. Movement and speech disorders have never previously been described and could be new features of the neurological phenotype.

## Introduction

Phosphatase and actin regulators (PHACTRs) are highly conserved proteins expressed in neuronal and non-neuronal cells (1). PHACTRs regulate cell morphology and motility during brain development (2) and modulate dendritic and axonal sprouting (1, 3).

*PHACTR1* is a modulator of angiogenesis, and it is implicated in coronary artery diseases and early-onset myocardial infarction (2, 4–6). It is also expressed in the rat (1) and mouse (7) brains, especially in the cortex, hippocampus, and striatum (1) and in pre- and post-synaptic dendrites during cortical development (7). The expression of the mutations in mice, through a dominant-negative manner, impairs developing brain neuronal migration, leading to mutant pyramidal cells that are misplaced and show abnormal morphology and polarity. These alterations were also seen in post-natal life, suggesting that PHACTR1 plays a role in post-natal brain maturation too (8).

*PHACTR1* variants have been explored in cardiovascular disease, but little is known about their role in neurodevelopmental disorders. In the literature, *PHACTR1* pathogenic variants have been described in six patients with neurological features, including infantile epilepsy with severe psychomotor delay (9, 10), early-onset West syndrome (8), and (in one case also carrying a mutation in *CPT1B*) severe intellectual disability, attention deficit hyperactivity disorder, and inability to stand or walk (11). Cardiological problems were not reported in any of these patients.

We here describe two unrelated patients with *de novo* novel missense pathogenic variants in *PHACTR1*, focusing on their neurological features.

## Cases

Patient 1 is a 17-year-old female, the second child of healthy non-consanguineous parents. She was born at term and the perinatal period was unremarkable. Psychomotor development was severely delayed with crawling reached at 2 years of age and walking starting at 3 years, with a rigid lower limb gait pattern. At 3 years, she was able to pronounce two words but subsequently lost this ability. Currently, she has a severe intellectual disability and communicates through guttural sounds, gaze, and a limited number of meaningful signs. She understands simple instructions.

**Figure 1:**
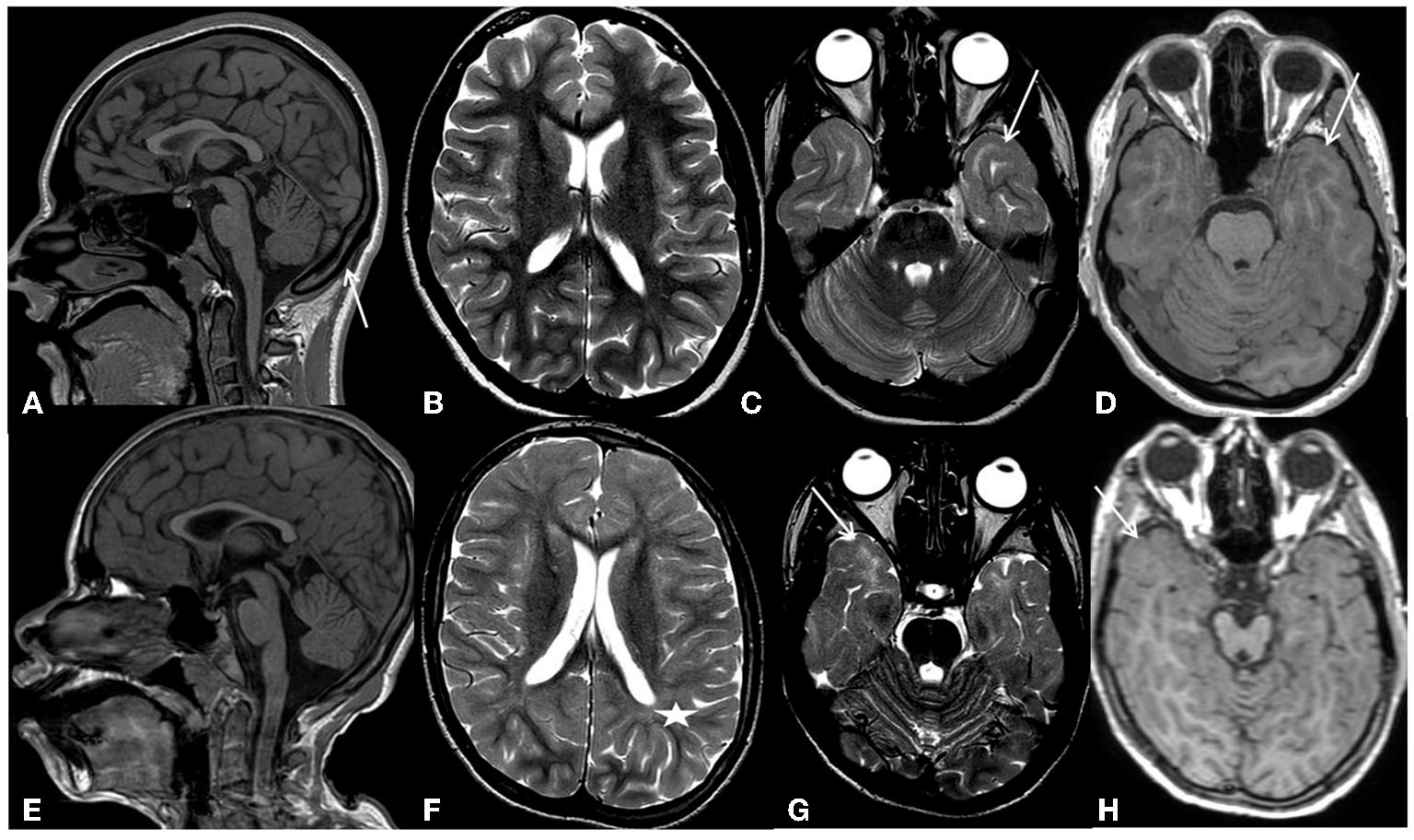
Patients' most recent MRI examinations. First row-Pt1: **(A)** (sagittal 3D T1-weighted image) shows thinning of the corpus callosum, reflecting brain volume reduction, and compensatory thickening of the skull (arrow). **(B)** (axial T2 weighted image) shows an overall slight reduction in white matter volume with moderately enlarged subarachnoid spaces. **(C)** (axial T2 weighted image), at the level of temporal poles, shows subcortical white matter signal hyperintensity (arrows). **(D)** (axial 3D-T1 weighted image), at the same level, shows a normal signal intensity of the subcortical white matter (arrow). Second row-Pt2: **(E)** (sagittal 3D T1-weighted image) shows microcephaly with thinning of the corpus callosum reflecting brain volume reduction. **(F)** (axial T2 weighted images) shows a diffuse reduction in white matter volume with moderate enlargement of the lateral ventricles and subarachnoid spaces. Diffuse blurring of the white matter signal is also evident in both hemispheres (asterisks). **(G)** (axial T2 weighted images), at the level of temporal poles, shows subcortical white matter signal hyperintensity (arrows). **(H)** (axial 3D-T1 weighted image), at the same level, shows a normal signal intensity of the subcortical white matter (arrow).

Epilepsy appeared at the age of 2 months, with seizures characterized by fixation of gaze, perioral cyanosis, and hypotonia, followed by asymmetric spasms in clusters. EEG showed a poor organization of background activity with high-frequency multi-focal epileptic discharges. The patient was diagnosed with early-onset encephalopathy with spasms. Treatment with valproic acid, benzodiazepines, and ACTH was started, achieving a partial response. In the course of her childhood, she presented focal seizures, sometimes with focal to bilateral tonic-clonic evolution and spasms. Several antiseizure medications (vigabatrin, hydrocortisone, and benzodiazepines) were tried, without obtaining seizure control or clear EEG improvement, until the age of 10 years, when the patient started treatment with lamotrigine. Since then, she has been seizure-free. From the age of 5 years, the patient showed a rigid-bradykinetic movement disorder with a progressive course. She presented mixed hypertonus, hypo-bradykinesia, and hypomimia and became increasingly unstable, with ataxic gait and retropulsion. Over time, she also experienced freezing gait and festination, which could be broken only by tactile stimulation and oculomotor apraxia. L-dopa up to 10 mg/kg/day was not effective. At approximately 10 years of age, she lost the ability to walk independently. Brain MRI was normal at 3 months of age, but at 2 years, it showed corpus callosum and bihemispheric white matter thinning as well as midbrain hypoplasia. At 8 years of age, MRI findings were basically unchanged, and a diffuse subcortical T2w signal hyperintensity involving both temporal poles was still evident in the first examination, suggesting an incomplete myelination process/myelination defect. At the last evaluation at the age of 16 years, brain MRI showed moderate brain atrophy (Figure 1). The subcortical T2w signal hyperintensity involving both temporal poles was still evident.

Laboratory analysis and electrophysiological studies at the age of 16 years were normal. Chromosomal karyotype, array-CGH, and NGS panel for Rett and epileptic syndromes were normal. Trio (proband and parents) whole-exome sequencing (trio-WES) identified a *de novo* heterozygous variant (NM_030948.6:c.1529T>G, p.Val510Gly) in the *PHACTR1* gene.

Currently, her cardiological investigations remain normal.

Patient 2 is a 14-year-old male, the first child of healthy non-consanguineous parents, born at term. With the exception of the finding of oligohydramnios, the pregnancy and perinatal period were uneventful. Psychomotor development was severely delayed; the patient walked with help at the age of 2 years. Language has never been acquired, and he showed severe intellectual disability.

At 3 months of age, the patient displayed focal spasms, and multi-focal epileptic discharges were observed on EEG, mainly in the right central regions. A diagnosis of atypical West syndrome was made. He was treated with vigabatrin and ACTH without success. Over the years, he presented focal seizures characterized by oromandibular automatisms, stiffness of the upper limbs, and loss of head control. Multiple drugs have been tried, such as phenobarbital, valproic acid, topiramate, lamotrigine, nitrazepam, and fenfluramine, without ever achieving seizure freedom.

The patient currently presented acquired microcephaly and diffuse severe hypotonia with normal reflexes. He could only walk for short distances with support, and he presented a severe but stable clinical picture.

At the age of 4 months, the brain MRI was normal. At 12 years, it showed a diffuse reduction of the white matter, especially in the frontal and temporal-polar lobes, and thinning of the corpus callosum and brainstem. A slight but diffuse blurring of the T2w signal within the bihemispheric white matter was observed, indicating a widespread incomplete myelination process/myelination defect; in particular, the temporal poles continue to show subcortical T2w signal hyperintensity. The MR spectroscopy study documented a reduction of the NAA peak with the inversion of the NAA/Ch ratio. At 14 years, the brain MRI showed a slight expansion of the supratentorial cortical spaces, indicating brain atrophy progression (Figure 1). A diffuse myeline signal alteration on T2 weighted images was still present, especially at the temporal lobe.

Laboratory analysis, chromosomal karyotype, array-CGH, and NGS for epilepsy genes were normal. Trio-WES then identified a *de novo* heterozygous variant (NM_030948.6:c.1553T>C, p.Ile518Thr) in the *PHACTR1* gene.

Currently, his cardiological examinations remain normal.

### Genetic analysis

The genome's exonic regions and flanking splice junctions were captured using the Clinical Research Exome v.2 kit (Agilent Technologies, Santa Clara, CA). Sequencing was done on a NextSeq500 Illumina system with 150 bp paired-end reads. The reads were aligned to human genome build GRCh37/UCSC hg19 and analyzed for sequence variants using a custom-developed analysis tool (12). Additional sequencing technology and a variant interpretation protocol have previously been described elsewhere (12).

Coverage on target for case 1 was ≥10x for 98.2% with a mean coverage of 210x, and for case 2, it was ≥10x for 87.4% with a mean coverage of 223x.

Both variants of the *PHACTR1* gene are in exon 13; in particular, the variant NM_030948.6: c.1553T> C, p.Ile518Thr affects the same base as the previously described mutation NM_030948.6: c.1553T> A, p.Ile518Asn (8). Variant p.Ile518Thr has been classified as probably pathogenetic (class 4 of the ACMG-American College of Medical Genetics); it is not described in the literature, and it is not present in gnomAD (MAF 0). Variant p.Val510Gly has never been described in the literature; it is disease-causative and is not present in gnomAD (MAF 0).

De-identified patient data are available upon request to the corresponding author.

## Discussion

We here described two previously unreported patients with *de novo* missense pathogenic variants in *PHACTR1* and compared them with cases reported in the literature.

Severe developmental delay, intellectual disability, and early-onset epilepsy, observed in our cases, seem to be among the main features of *PHACTR1*-mutated patients with neurological involvement (Table 1). Instead, communication deficits and, in particular, the specific involvement of expressive language and speech observed in patient 1 have never been previously described. Similar to four of the six previously reported patients, our patients presented with spasms in the first months of life and received a diagnosis of West syndrome (8, 10). Despite the literature cases being diagnosed as West syndrome, we now refer to this condition as an early-onset developmental and epileptic encephalopathy, as stated in OMIM. Although the majority of reported cases have drug-resistant seizures, our patient 1 became seizure-free on lamotrigine.

**Table 1:** Review of *PHACTR1*-mutated cases reported in the literature including the current cases.

**Patient**	**Sex**	***PHACTR1* variants - inheritance**	**Prediction software**	**Epilepsy**	**Drug resistance**	**Neuropsychiatric problems**	**Additional features**	**Brain MRI**
De Ligt et al. (9)	F	c.1561C>T p.(Arg521Cys) - *de novo*	Predicted pathogenic	Yes – onset at 3 weeks	No	DD, psychomotor regression, severe ID, spastic tetraparesis	Scoliosis, microcephaly, difficulty thrivinging	Not reported
The Deciphering Developmental Disorders Study	F	c.1553T>A p.(Ile518Asn) - *de novo*	Predicted pathogenic	Not reported	NA	DD	Not reported	Not reported
Hamada et al. (8)	M	c.1499T>C p.(Leu500Pro) - *de novo*	Predicted pathogenic	Yes – WS at 3 months. Epileptic spasms and tonic seizures	Yes	DD, severe ID, hypotonia	Polyhydramnios, small for gestational age (SGA), cryptorchidism	Repeat brain MRIs at 3 months and 20 months of age revealed progressive atrophy and delayed myelination
	M	c.1436A>T p.(Asn479Ile) - *de novo*	Predicted pathogenic	Yes – WS at 3 months. Epileptic spasms and tonic seizures	No	DD, severe ID, autism, paroxysmal upward gazing	Facial dysmorphism: short eyebrows, fullness of upper eyelids, small mouth, widely spaced eyes, low-set ears	Normal
Riazudin et al. (11)	uk	c.1148C>T p.(Ser383Leu) - *de novo* Also, mutation c.2048G4A/ p.(Arg683His) in *CPT1B*	Predicted pathogenic	No	NA	Severe ID, ADHD, speech delay, severe hypotonia, inability to hold head, stand or walk	Not reported	Not reported
Marakhonov et al. (10)	M	c.1556T>G p.(Leu519Arg) - *de novo*	Predicted pathogenic	Yes – epileptic spasms and focal motor seizures evolving into bilateral tonic-clonic seizures from age 3 months	Yes	DD, generalized hypotonia	Not reported	At 4.5 m.o. hypoplasia of the corpus callosum, non-obstructive external hydrocephalus and delayed myelination. At 6 m.o. no changes
Patient 1	F	c.1529T>G p.(Val.510Gly)- *de novo*	Predicted pathogenic	Yes – epileptic spasms and focal seizures from age 2 months	Yes – now seizure-free	DD, psychomotor regression, severe ID, rigid-bradykinetic movement disorder	Mild facial dysmorphisms: deep-set eyes, long eyelashes	Moderate midbrain hypoplasia, diffuse thinning of the corpus callosum and mild delay in myelinization of the white matter of the cerebral hemispheres
Patient 2	M	c.1553T>C p.(Ile518Thr) - *de novo*	Predicted pathogenic	Yes – epileptic spasms and focal seizures from age 3 months	Yes	Severe ID, acquired microcephaly, not able to walk	Mild facial dysmorphisms: deep-set eyes, downslanting eyelids, flat nasal root, low-set ears, fullness of the upper lip, large mouth, hypothyroidism	Diffuse reduction of white matter, especially in frontal and temporo-polar lobes; thinning of the corpus callosum and brainstem.

F, female, M, male; WS, West syndrome; DD, developmental delay; ID, intellectual disability; ADHD, attention deficit hyperactivity disorder; m.o., months of age; uk, unknown; N.A, not applicable.

**Figure 2:**
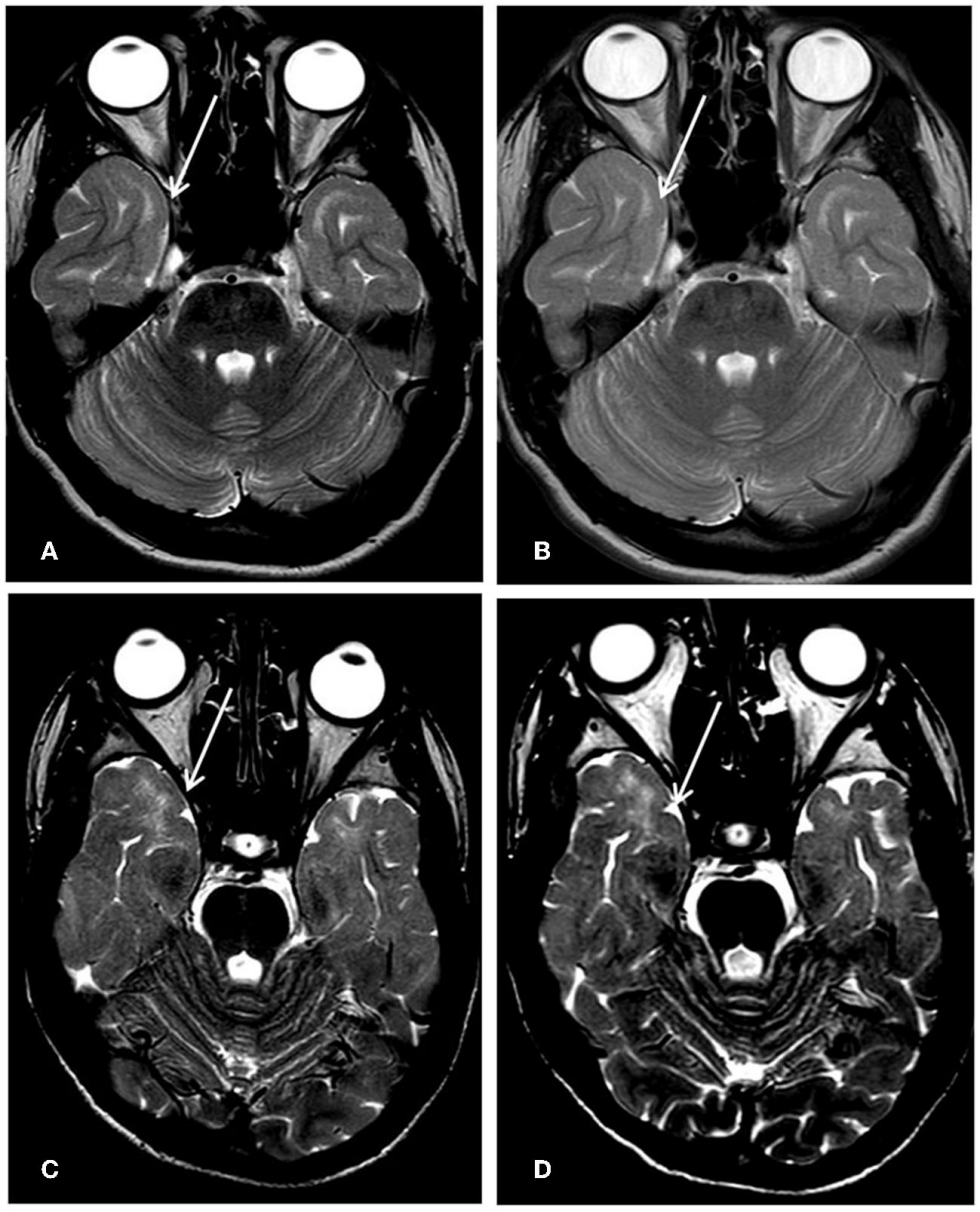
First row Pt1, 8-year-old's MRI **(A)** vs. 16-year-old's follow-up **(B)**; second row Pt2, 12-year-old's MRI; **(C)** and 14-year-old's follow-up **(D)**. The subcortical white matter signal hyperintensity on T2 weighted axial images at the level of temporal poles (arrows) resulted unchanged during the time.

The brain MRI findings in our patients support the idea that *PHACTR1* mutations can be associated with slightly progressive encephalopathy. Our long-term neuroimaging follow-up showed progressive brain atrophy and persisting diffuse subcortical T2w signal hyperintensity of the temporal poles, indicating an incomplete myelination maturation/myelin defect. Although temporal pole subcortical areas usually do myelinate in the later phases of the process, Parazzini et al. (13) demonstrated that the process of myelination is completed by 40 months of age. On the contrary, Maricich et al. (14), in their cohort, demonstrated that myelination of cortical areas was not complete at 46 months of age. However, in our cases, the T2w signal hyperintensity of the temporal poles persisted during the time, even at the teenage MRI control (Figure 2), confirming a myelination defect rather than delayed myelination. The myelination defect together with the brain atrophy represents unspecific MRI phenotypical features of the genetic defect. Similar findings have been described in early epileptic encephalopathies associated with *STXBP1* pathogenic variants (15). Anterior temporal lobe T2 white matter hyperintensity has also been described in other encephalopathies such as vascular diseases (cerebral autosomal dominant/recessive arteriopathy with subcortical infarcts and leucoencephalopathy-CADASIL/CARASIIL, respectively), infection diseases (congenital cytomegalovirus infection), dysmyelinating diseases (megalencephalic leukoencephalopathy with subcortical cysts-MLC), and neuro-degenerative diseases (Aicardi-Gutierres syndrome) (16). CADASIL/CARASIL is a progressive neurodegenerative condition with a rare but possible childhood onset. The subcortical anterior temporal involvement represents a specific neuroradiological characteristic, together with the involvement of the external capsule and the coexistence of lacunar infarcts and microbleeding; in this condition, the temporal findings represent areas of “leukoarariosis,” indicating a degenerative leukopathy related to micro-vessels disease and consisting of white matter rarefaction/gliosis with numerous fluid-filled perivascular spaces (PVS) or increased number of PVS in the cortex (17). In the other conditions, the temporal lobe T2 hyperintensity is usually associated with temporal cysts, rather than indicating destroying parenchymal phenomena; in the MLC temporal lobe, swelling also coexisted due to intramyelin edema. Moreover, in all these conditions, the T2 signal was higher than in *PHACTR1* temporal lobe alteration, and in T1 weighted images, the signal results were low; on the contrary, in our patients' condition, T1-weighted images showed a normal signal at that level, as seen in hypomyelination disorders (Figures 1D, H).

Myelination is one of the most important factors influencing brain maturation and epileptogenesis (18). Our two cases confirm that *PHACTR1* plays a role in post-natal brain maturation (8). Until now, no other distinct MRI features can be ascribed to the syndrome even though non-specific alterations of the corpus callosum have been observed in one individual (11).

The cardiological follow-up did not show any abnormalities in our patients; however, considering that in the described cases, cardiological involvement arises later in life, we cannot rule out that a long-term follow-up should be carried on.

As reported herein, our patient 1 developed a movement disorder consisting of mixed hypertonus, hypo-bradykinesia, hypomimia, ataxic gait with retropulsion, freezing of gait, festination, and oculomotor apraxia. Movement disorder has never previously been described in *PHACTR1-*mutated patients. Although the gene is known to be highly expressed in cortical neurons, some studies have also found *PHACTR1* expression in the basal ganglia, particularly the caudate putamen, in both the developing and the adult mouse brain (19). Notably, Wider et al. (20) found an association between a single nucleotide polymorphism in *PHACTR2* and Parkinson's disease; this association is yet to be fully investigated and clarified, but it could provide clues about the role of PHACTRs in movement disorders. These authors' findings highlight the need for a clearer and more specific understanding of the role of *PHACTR1* in the regulation of the extrapyramidal system.

## Conclusion

*PHACTR1* is reported as a genetic susceptibility locus as part of a complex genetic pattern of inheritance in cardiovascular diseases; moreover, in those patients, no neurological involvement is described. Conversely, the few cases with neurological problems showed no cardiac abnormalities. This suggests that different mutations in *PHACTR1* may result in two different phenotypes, one cardiological and the other neurological, which do not overlap in the same patient. So far, no direct genotype-phenotype correlations have been found in the described cases.

The core phenotype of *PHACTR1* mutation consists of severe developmental delay/intellectual disability and epilepsy. Our novel report of a *PHACTR1-*mutated patient presenting with a rigid-bradykinetic movement disorder, therefore, expands the phenotypic spectrum. Further studies are needed to assess whether this movement disorder should be classified as a different phenotype or an evolution of the disease. The different clinical presentations of the cases reported underline that gene mutations can show great phenotypic heterogeneity, making further clinical and biochemical studies necessary to identify the underlying pathophysiological mechanisms.

## Data availability statement

The datasets presented in this article are not readily available because of ethical and privacy restrictions. Requests to access the datasets should be directed to the corresponding author.

## Ethics statement

Ethical review and approval was not required for the study on human participants in accordance with the local legislation and institutional requirements. Written informed consent to participate in this study was provided by the participants' legal guardian/next of kin.

## Author contributions

AL and RP wrote the first draft and performed data curation. GI performed the formal neuroradiological analysis. CS, AL, RP, and MB performed clinical data curation. MI performed genetic data curation. SB was responsible for the conceptualization and supervision of the study. AL, RP, MB, GI, CS, LS, MI, PV, and SB reviewed and approved the manuscript before submission. All authors contributed to the article and approved the submitted version.
